# Sharing with More Caring: Coordinating and Improving the Ethical Governance of Data and Biomaterials Obtained from Children

**DOI:** 10.1371/journal.pone.0130527

**Published:** 2015-07-01

**Authors:** Holly Longstaff, Vera Khramova, Elodie Portales-Casamar, Judy Illes

**Affiliations:** 1 National Core for Neuroethics, Division of Neurology, Department of Medicine, University of British Columbia, Vancouver, British Columbia, Canada; 2 Centre for Molecular Medicine and Therapeutics at the Child and Family Research Institute, University of British Columbia, Vancouver, British Columbia, Canada; Pasteur Institute of Iran, ISLAMIC REPUBLIC OF IRAN

## Abstract

**Introduction:**

Research on complex health conditions such as neurodevelopmental disorders increasingly relies on large-scale research and clinical studies that would benefit from data sharing initiatives. Organizations that share data stand to maximize the efficiency of invested research dollars, expedite research findings, minimize the burden on the patient community, and increase citation rates of publications associated with the data.

**Objective:**

This study examined ethics and governance information on websites of databases involving neurodevelopmental disorders to determine the availability of information on key factors crucial for comprehension of, and trust and participation in such initiatives.

**Methods:**

We identified relevant databases identified using online keyword searches. Two researchers reviewed each of the websites and identified thematic content using principles from grounded theory. The content for each organization was interrogated using the gap analysis method.

**Results:**

Sixteen websites from data sharing organizations met our inclusion criteria. Information about types of data and tissues stored, data access requirements and procedures, and protections for confidentiality were significantly addressed by data sharing organizations. However, special considerations for minors (absent from 63%), controls to check if data and tissues are being submitted (absent from 81%), disaster recovery plans (absent from 81%), and discussions of incidental findings (absent from 88%) emerged as major gaps in thematic website content. When present, content pertaining to special considerations for youth, along with other ethics guidelines and requirements, were scattered throughout the websites or available only from associated documents accessed through live links.

**Conclusion:**

The complexities of sharing data acquired from children and adolescents will only increase with advances in genomic and neuro science. Our findings suggest that there is a need to improve the consistency, depth and accessibility of governance and policies on which these collaborations can lean specifically for vulnerable young populations.

## Introduction

The dramatic rise in collaborations using databases and biobanks over the past decade underscores the hopes of research sharers and those who access research from around the world about these new capabilities and the novel medical discoveries that they can foster [1,2]. These efforts recently culminated in the *Global Alliance to Enable Responsible Sharing of Genomic and Clinical Data* [3] a new initiative involving over seventy international organizations interested in sharing genomic and clinical data. Many data sharing organizations, like the Global Alliance, are now recruiting data contributors and users online, outside the face-to-face presence of a trusted medical professional. Examples of other organizations engaged in either on or offline recruiting include (1) the International Cancer Genome Consortium (ICGC); (2) databases governed by funding agencies such as the NIH and CIHR; (3) public, non-profits, and advocacy organizations such as Autism Speaks; and (4) databases driven by patients who directly submit their own health information and history online such as patientslikeme. These organizations allow multiple researchers to access previously submitted data in order to conduct novel analyses. These efforts are intended to maximize the efficiency of invested research dollars [4], increase the ease of data access [5], minimize the costs of new recruitment and consent procedures to researchers [6], reduce the participatory burden on research participants [7], increase citation rates of publications associated with data analyses [8], and expedite research outputs and benefits to patients [9].

Despite these benefits, data sharing may also lead to a variety of potential risks for participants. Private medical information is frequently the source of data entered and accessed in these databases. Hence, it is crucial that information about the ethical governance mechanisms established by central institutions is transparent and easily available to prospective contributors. Adding to the complexity of the issue is the lack of harmonization across databases and biobanks for types of data stored, data entry formats, and policies for protecting the privacy and confidentiality of contributor populations, especially for highly vulnerable groups such as children and youth [10]. Navigating and appreciating nuances associated with each unique set of norms for good decision-making, therefore, can be a circuitous exercise [11].

Minors are widely recognized as potentially vulnerable research participants because they lack the legal and, at some ages, also the cognitive capacity to provide informed consent to research [12]. While children are unable to provide informed consent to research participation, their expression of assent must be carefully considered according to ethics guidance documents in Canada (Article 3.10 TCPS2) and the US (Subpart D of 45CFR46). In the case of research on neurodevelopmental disorders, children may be considered especially vulnerable due to variability in behavioral and cognitive functioning, stigma associated with their conditions, and long-term risks to privacy and confidentiality associated with central resources that may have extremely long operational timeframes and have the potential to harm the child well into adulthood. In this study, we focus on current resources and strategies for data sharing involving children representing three of the most common neurodevelopmental disorders in the Western World: autism spectrum disorder (ASD), cerebral palsy (CP), and fetal alcohol spectrum disorder (FASD). The British Medical Association reports that in the west, FASD is the leading cause of non-genetic intellectual disability, and the US Centers for Disease Control and Prevention states that CP affects between 1.5 and 4 per 1000 children from around the world, while ASD is believed to affect tens of millions of children worldwide according to Autism Speaks. In combination, these disorders impact over one million minors in Canada. The heterogeneity of presentation in these neurodevelopmental disorders [13–15] creates the need for large study sample sizes with sufficient power to discern small but significant physiological effects [16], many of which evolve over time. While shared databases are ideal for solving these experimental challenges, we hypothesized that the stewardship of data and biological tissues [17] and language about special protections for certain groups such as minors, would be variable and opaque given the rapid pace and diverse strategies with which many of these database resources have been built. These limitations in the ethical governance of data and biomaterials obtained from children have the potential to compromise the bidirectional trust relationship that is vital to engaging any donor community directly or through its surrogate [18, 19]. This phenomenon can be realized in a number of ways. In order to foster data sharing between databases, donors must be willing to contribute data, and this will not take place if individuals do not trust the data steward to protect, manage, and use their data in responsible ways. Instructive examples include the 2010 Texas case in which the blood spot samples of approximately five million infants were destroyed to address public outrage when materials were used for secondary purposes without the informed consent of donors [20], and the 2013 Gymrek case [21] in which participant surnames were uncovered when researchers were able to breach the anonymity of genetic databases. These examples of unethical use and inadequate governance mechanisms can erode public trust and support for databases, and thereby limit the ability of databases to produce meaningful results. The phenomenon is compounded when there is a lack of readily available information about data sharing procedures and ethical governance that delays access to data by researchers. Our goal was therefore to characterize the strengths of these centralized resources for biomaterials dedicated to children, identify gaps, and to use the results to deliver evidence-based recommendations for improving mechanisms for their proactive ethical governance.

## Methods

We carried out an online search, thematic analysis and a gap analysis [22, 23]. The overarching objective of the study was to derive patterns about the type of information that is and ought to be presented on these websites, not to evaluate individual websites per se.

### Search Strategy

We used online search engines such as Google to identify relevant websites for analysis. Sites were identified using the following key words and configurations:

1. {condition}{acronym}{data sharing}{database}
2. {country}{submitting disorder data to database}{acronym}
3. {country}{condition}{acronym}{registry}
4. {country}{condition}{acronym}{register}

Searches were run sequentially for each of three conditions of interest–ASD, CP and FASD–and three regions of interest for this study: Canada, United States (USA), and United Kingdom (UK). The first ten pages of search results were analyzed in order to identify relevant organizations. To be considered for analysis, organizations had to provide materials written in English; be based in the target countries; share unique data or biomaterials; and hold data primarily collected from children and youth with ASD, CP, or FASD. Searches were conducted over the period between July 2012 and April 2014, and were replicated in October 2014 to verify results from the previous year. Only minor changes were noted during the second search and these results are reflected in the tables presented. Experts in the field of neurodevelopmental disorders research reviewed the complete sample to confirm eligibility and comprehensiveness. We note that Google searches may not be replicable and results from similar future searches will vary from user to user.

### Thematic Analysis

Two independent researchers trained in qualitative methods reviewed each of the websites included on the expert vetted list, and amassed a list of emergent ethics themes using principles from grounded theory [24]. This interactive coding method begins with an initial, open coding phase followed by a more focused round of coding and is conducted without a quantitative coding frame informed by preconceived notions from literature or expert opinion [25]. After the coding book was constructed, additional themes were added in consultation with experts in the field of research ethics to ensure completeness. This master list of themes comprise many of the topics enshrined in national research ethics policies such as *45 CFR part 46* in the United States and the *Tri-Council Policy Statement*: *Ethical Conduct for Research Involving Humans (TCPS2 2014)* in Canada. Particular themes such as confidentiality, the informed consent/assent relationship, benefit sharing, commercialization, and incidental findings certainly receive a greater share of attention in the peer reviewed literature due to controversy amongst experts regarding how they ought to be operationalized and potential harms to human research participants associated with each theme. However, we believe that we are justified in including all emergent themes in our analysis regardless of their significance in the literature since all, to a lesser or greater extent, are an integral part of good governance strategies for data sharing and biobanking endeavors.

The overall list of themes was then divided into two sub-categories: (1) procedural and (2) substantive ethics themes. Procedural ethics can be described as ethically constructed strategies and fair procedures used in research, such as properly constructed consent forms [26]. Substantive ethics are understood as the ethical or moral values and objectives that guide research, such as preventing harms to research participants or avoiding stigmatization of participant groups [27]. Attention must be paid to both ethics concepts in order to achieve just outcomes in health research [an extensive overview of substantive and procedural issues in health-related topics can be found in [28]].

### Gap Analysis

The website content from each organization was reviewed to determine convergences and gaps in emergent themes. This method interrogates documents for major themes and subthemes, and is particularly suitable for the analysis of heterogeneous sets of documents or materials typically found in online resources such as websites [22, 23, 29, 30,31]. Using this method, documents are coded independently and rigorously by each researcher for the significant or brief presence of an identified theme compared to the other documents in the sample (as opposed to an external standard in the field of research ethics). *Disagreements between the trained coders are resolved through deliberation and negotiation* [32, 33, 34]. If a theme does not appear in any meaningful way in the document then it is registered as a gap. Determination of significance and brevity is subjective, involving measurements that depend on a shared understanding of complex ethics themes. Deliberation leading to consensus between the trained coders is essential throughout the coding process. Even with considerable pre-coding discussions, disagreements are inevitable and each instance, therefore, was carefully analyzed. The researchers reviewed the materials after the first analytic iteration and deliberated on any discrepancies until 100% consensus on each code was achieved. The senior author was available to adjudicate any issues that could not be resolved this way between the two coders, but in no case was this necessary. Gap analysis results here reflect website content as of October 2014.

## Results

The websites analyzed in this study are highly specialized and therefore limited in number. Our search revealed twenty-three databases that addressed our topic area, but only sixteen met our inclusion criteria resulting in a relatively small yet homogeneous sample. Of these, eleven were focused on ASD (four in the USA, two in Canada, three in multiple locations, and two in the UK), four focused on CP (one in the USA, one in Canada, two in the UK), and only one was focused on FASD. Subsequent content analysis of these websites revealed eleven themes that could be characterized as primarily procedural (see Table 1) and four that could be characterized as mostly substantive in nature (see Table 2). Themes identified as procedural included factually based information about the data (i.e., type, volume), and requirements, guidelines, and protocols about how to use the data. Themes identified as substantive included database information addressing ethical concerns such as special considerations that may be required for minors, the handling of incidental findings, commercialization issues, and ways to promote benefit sharing.

**Table 1:** Procedural ethics themes and examples.

Procedural ethics themes	Examples of information encompassed by theme
1 Types of data and tissues stored	*Included types of genotypic information*, *behavioral questionnaires*, *biosamples*
2 Accessibility	*Structure of access (restricted*, *open*, *tiered)*, *methods of controlling access*
3 Requirements for permission to access data and tissues	*Application to the organization*, *proof of Institutional Review Board (IRB)/Research Ethics Board (REB) approval*
4 Confidentiality	*Protection of privacy*, *methods of de- or re- identification*, *encryption systems*
5 Data quality control	*Verification of data protocols*, *stated standards for submission*
6 Consent/assent guidelines	*Guidelines for re-consenting*, *allowance for secondary use of data*
7 Volume of data and tissues stored	*Number of samples*, *participants*, *contributors*
8 Data management/updating	*Permissions to update data*, *protocols for removal of data*
9 Requirements to store data and tissues	*Permitted data submitters*, *data formats accepted*
10 Control to check if data/tissues are being submitted	*Presence of verification if data is being submitted*, *frequency of submission checks*
11 Disaster recovery	*Protocols for system crashes*, *plans for shutting down of database*, *data back-ups*

**Table 2:** Substantive ethics themes and examples.

Substantive ethics themes	Examples of information encompassed by theme
Benefit sharing	*Remuneration*, *therapeutic benefits*, *return of individual results or overall research findings*
Commercial ties	*Data rights*, *plans for monetary collection/distribution*
Special considerations for minors	*Assent models*, *return of results to minors*, *protocols for included minors reaching majority*
Incidental findings (IFs)	*Protocols for IFs of clinical significance*, *IFs with no clinical significance*, *further professionals to consult on potential IFs*

### Gap Analysis Results

Table 3 presents the gap analysis results according to each procedural and substantive ethics theme. As the independence of the data cannot be established, quantitative comparisons are not suitable for this analysis. Instead, we report the data in the form of descriptive statistics. In this Table, a 0% gap indicates that all websites analyzed for this study discussed this theme in either a significant or brief way, while a 100% gap would indicate that the theme was not mentioned at all on any of the websites.

**Table 3:** Gap analysis results.

Procedural ethics theme	Significant	Brief	Gap
Data and tissues stored	88%	13%	0%
Accessibility	63%	19%	19%
Requirements for permission to access data and tissues	56%	31%	13%
Confidentiality	56%	31%	13%
Volume of data and tissues stored	56%	6%	38%
Data quality control	50%	19%	31%
Consent/assent guidelines	44%	38%	19%
Data management/ updating	38%	38%	25%
Requirements to store data and tissues	25%	38%	38%
Control to check if data/tissues are being submitted	6%	13%	81%
Disaster recovery	6%	13%	81%
**Substantive ethics theme**	**Significant**	**Brief**	**Gap**
Benefit sharing	44%	31%	25%
Commercial ties	25%	31%	44%
Special considerations for minors	13%	25%	63%
Incidental findings (IFs)	6%	6%	88%

Totals may not equal 100% due to rounding error.

### Procedural Ethics Themes

The procedural theme most commonly addressed in a significant way by fourteen organizational websites was *data and tissues stored* (88%). One illustrative example is a database on which there are lists of behavioral data questionnaires. The website for the database also includes text stating that the organization collects diagnostic information, medical history, and information on special educational needs of the participating individuals. The second most common procedural theme addressed in a significant way by 63% of the organizational websites was *accessibility*. A majority of organizations that discussed the topic state that any researchers who are approved for access will have all database data available to them. Some, however, grant tiered access for more sensitive or more easily identifiable data, requiring an additional round of applications. Three websites (19%) cover this theme in a brief manner, for example, as outlined in a passage from another website, which states that eligible researchers have access to datasets housed there.

More than half (56%) of the websites reviewed for this study addressed the *requirements for permission to access data and tissue confidentiality* concerns, and *volume of data and tissues stored* in a significant manner, a few addressed them in a brief way. In the case of permission requirements, application forms were posted directly on the websites and were easily downloadable for some organizations, while others clearly stated their access requirements. When present, the applications were often quite rudimentary, however, requiring the requester’s name, affiliation, principal investigator on the relevant project, and an IRB/REB number. Little information was present regarding criteria for accepting applicants or for what sorts of applications would be accepted or denied, or on what grounds such decisions were being justified. Examples of how *confidentiality* was addressed include descriptions of encryption systems and measures employed, details of separate storage methods for identifiable data and codes for de-identified data, and information on database storage logistics.

The next most common procedural theme was *data quality control* (e.g., standardized forms for data submission. This was addressed significantly in half of the websites reviewed (50%). *Consent/assent guidelines* were addressed in a significant way in less than half of the websites (44%), while *data management and/or updating plans* were entirely absent in one quarter (25%) of the sites.

Descriptions of *requirements for data and tissue storage* were entirely absent from six (38%) of the websites. When they were mentioned, these requirements included lists of participating university medical clinics allowed to contribute data and data collecting tools allowed to foster standardization of data submitted from contributing researchers. Brief references on six (38%) of the websites constitute simple statements such as the requirement for physician data entry.

Procedural themes that were least likely to be addressed, or emerged as the largest gaps, included *controls to checking data/tissues are being submitted* (gap for 81% of websites) and *disaster recovery* (gap for 81%).

### Substantive Ethics Themes

*Benefit sharing* was the most commonly addressed substantive theme in 44% of the websites and the most common method of addressing benefit sharing was through online newsletters. *Commercial ties* were addressed significantly in one quarter (25%) of the online resources while *special considerations for minors* was only significantly presented on 13% of websites. We found that *incidental findings* (IF) was the substantive theme least likely to be discussed on any of the websites. This theme was not addressed in fourteen (88%) of the websites reviewed for this study and therefore represents a major informational gap. When IF was discussed, the website typically advised that adults can decide whether or not to be informed of any incidental genetic findings of clinical significance for them or their child.

## Discussion

A review of policies, established by major health research funding bodies in the USA, Canada and the UK, demonstrates that there is significant support for data sharing to promote public good. All the policies summarized in Table 4, for example, are strongly supportive of data sharing efforts and this tone is echoed in the policies and requirements of high impact scientific journals such as *Science*, *Cell*, and *Nature*. It is perhaps unsurprising then that databases funded through public and other granting structures and whose scientific advancements are announced and shared through peer reviewed journals are also embracing the open data movement. Yet while these efforts are viewed as a means to serve the public good, this review of international organizations interested in sharing genomic and clinical data for neurodevelopmental research demonstrates that much work remains to be done to foster the ethical governance of data in order to protect the rights of donors.

**Table 4:** Examples of data sharing policies.

Funding Agency	Data sharing policy excerpt
Genome Canada Data Release and Resource Sharing	*Genome Canada is committed to the principle of rapid data release and sharing of unique resources to the scientific community; Genome Canada-funded projects must therefore share data and resources in a timely fashion with minimal or no restrictions*. *By providing the scientific community with timely access to the outputs of Genome Canada-funded projects*, *this data and resource sharing policy is intended to accelerate the translation of research for the benefit of humankind*. Retrieved February 10, 2014 from: http://www.genomecanada.ca/medias/PDF/EN/DataReleaseandResourceSharingPolicy.pdf
Canadian Institutes of Health Research Open Access Policy	*Recognizing that access to research data promotes the advancement of science and further high-quality and ethical investigation*, *CIHR explored current best practices and standards related to the deposition of publication-related data in openly accessible databases*. *As a first step*, *CIHR will now require grant recipients to deposit bioinformatics*, *atomic*, *and molecular coordinate data into the appropriate public database*, *as already required by most journals*, *immediately upon publication of research results (e*.*g*., *deposition of nucleic acid sequences into GenBank)*. Retrieved February 10, 2014 from: http://www.cihr-irsc.gc.ca/e/46068.html#8
Social Sciences & Humanities Research Council Research Data Archiving Policy	*SSHRC is committed to the principle that the various forms of research data collected with public funds belong in the public domain*. *Accordingly*, *SSHRC has adopted a policy to facilitate making data that has been collected with the help of SSHRC funds available to other researchers*. Retrieved February 10, 2014 from: http://www.sshrc-crsh.gc.ca/about-au_sujet/policies-politiques/statements-enonces/edata-donnees_electroniques-eng.aspx
National Institutes of Health	*Data sharing is essential for expedited translation of research results into knowledge*, *products and procedures to improve human health*. *The Final NIH Statement on Sharing Research Data was published in the NIH Guide on February 26*, *2003*. *This is an extension of NIH policy on sharing research resources*, *and reaffirms NIH support for the concept of data sharing*. Retrieved February 10, 2014 from: http://grants.nih.gov/grants/policy/data_sharing/
National Science Foundation	*Investigators are expected to share with other researchers*, *at no more than incremental cost and within a reasonable time*, *the primary data*, *samples*, *physical collections and other supporting materials created or gathered in the course of work under NSF grants*. *Grantees are expected to encourage and facilitate such sharing*. Retrieved February 10, 2014 from: http://www.nsf.gov/bfa/dias/policy/dmp.jsp
National Health Service, England	*Sharing information in the NHS helps ensure that the quality and safety of services is consistent across the country*. *It can also highlight different diseases and conditions that may require more NHS investment*. *The care*.*data programme gives an opportunity for each of us to help the NHS provide high quality care for all*. Retrieved April 2014 from: *http*:*//www*.*england*.*nhs*.*uk/ourwork/tsd/care-data/*

Of the sixteen websites examined for this study, only one focussed on FASD. This paucity represents a gap that should be addressed by the scientific research community given the prevalence of the condition in North America and the UK. Beyond this immediate gap, the results further reveal a lack of consistency across different databases and important gaps in ethical governance mechanisms and guidelines. The finding is especially troubling given the enhanced vulnerability of the donor population in this case as well as the study topic of neurodevelopmental disorders (ASD, CP, and FASD). The aim of this study was to replicate the kind of results that might emerge when potential donors search for databases. We were interested in analyzing the content of these websites to explore the ways in which they present ethics-related themes during their passive recruitment effort of participants. While it is possible that more information may be available for those who registered on these websites and actively began contributing their data, this review illustrates and examines the range of informational materials that are readily available to the lay public user and potential donors before a relationship is established with the database stewards. What is revealed in this initial review may have a significant impact on donors’ decisions to contribute data and also frames the subsequent informed consent process. The small, manageable, sample size of sixteen was established in order for the research team to conduct in–depth content analysis over time. The secondary search conducted in October 2014 demonstrates that the content of these websites did not change significantly from the previous year, and underscores the stability of these results.

Of the fifteen prominent themes that emerged in the analysis, only four could be properly characterized as primarily substantive by nature. This is perhaps unsurprising given that these themes are necessarily more difficult to operationalize than their procedural counterparts, which often focuses on compliance issues such as storage requirements and permissions.

Yet even the procedural themes such as *control to check if data or tissues are being submitted* or *disaster recovery* measures that in theory should be easy to address, were exposed as gaps in the analysis. This finding is significant as these themes represent low hanging fruit for data stewards. Gaps that emerged from the review of substantive ethics themes will undoubtedly be more difficult to conceptualize and tackle. In this case, only *benefit sharing* was addressed in any meaningful way while major gaps were revealed around the topic of *IF* (88% gap) and *special considerations for minors* (63% gap). As mentioned earlier in this paper, *benefit sharing* and *IF* (along with *commercialization*, *consent/assent guidelines*, and *confidentiality*) are perceived to be some of the most important and therefore problematized themes in this research ethics literature, which perhaps explains why they are more likely to be covered by websites in a significant or brief way. However, this also makes the gap associated with the *IF* theme even more compelling.

## Conclusions and Recommendations

Basic and clinical research can benefit greatly from the large scale sharing of data. However, to foster this effort, stakeholders must be satisfied that data sharing practices are ethically constructed and accountable to donor communities. This entails establishing additional safeguards and procedures for the protection of research participants that are particularly vulnerable to breaches of confidentiality, stigmatization, or other adverse consequences of data sharing. In this regard, some types of data must be handled differently to reduce the risk of harm to donors [34]. The significant gaps that emerged from our analysis around the topics of IF and considerations for minors are especially concerning given the recent focus on these important areas by for example, the Bioethics Commission in their 2013 report *Anticipate and Communicate*: *Ethical Management of Incidental and Secondary Findings in the Clinical*, *Research*, *and Direct-to-Consumer Contexts* [35], in the recent ACMG guidelines [36] and by the Panel on Research Ethics in the recent changes to *TCPS2 (2014)*[37] As explained by Wilfond and Carpenter [38], the decision context and ethical issues surrounding IF become even more complicated and difficult to navigate in the pediatric context. New procedures established in these areas will therefore depend on the nature of the data and participant population under study. Here, it would serve data sharing organizations well to seek out participant and stakeholder (i.e., advocacy groups and community advisory boards) perspectives to improve data sharing processes through the identification of potential unappreciated risks and benefits [39]. Children and families taking part in neurodevelopmental research may have a unique set of concerns and perspectives that could be used to inform gaps in these substantive ethical areas. This approach would also include calls for feedback regarding data sharing policies and the dissemination of information that would ensure that data sharing rationales are made accessible and understandable to donor communities [34]. Organizations that engage with stakeholders on this level and incorporate patient and community perspectives into data sharing policies will serve to foster ethical governance and public trust in their organization by ensuring respect for donors and the communities they represent.

The focus of this study was on the publicly available materials presented by organizations during the passive recruitment of human research participants. It may be the case that the internal information presented to interested participants who actually sign up to the websites is more robust. While this could be viewed as a limitation to this study, we assert that excluding vital information during all aspects of the recruitment process is a missed opportunity for organizations that wish to initiate a trusting and transparent relationship early on with participants. It may not be practical or even desirable to present all relevant information. However, we would suggest that a discussion of the following themes as a minimal set of criteria to include on all publicly available information given their significant focus in the bioethics literature and their importance to the protection of human research participants:

- Storage of data and tissue
- Accessibility
- Requirements for permission to access data and tissues
- Confidentiality
- Consent/assent guidelines
- Benefit sharing
- Commercialization
- Special consideration s for minors
- Management of incidental findings

Information concerning the above themes should be easy for potential participants to locate and navigate and could also include references to in-depth discussions and related topics found in internal documents for those who seek additional information. With minimal costs, organizations could succinctly summarize information concerning these themes in the publicly accessible areas of their websites and in the process enhance recruitment efforts while also informing individuals of the ethics issues relevant to research participation.
